# Biomimetic Porous Coatings on a Biocompatible Ti-15Mo Alloy as a Platform for Local Delivery of Anticancer Drugs to Patient Tissues

**DOI:** 10.3390/biomedicines13112779

**Published:** 2025-11-14

**Authors:** Svetlana Gatina, Ruzil Farrakhov, Alfiz Gareev, Azat Sabitov, Nariman A. Enikeev, Natalia Anisimova, Mikhail Kiselevskiy

**Affiliations:** 1 Laboratory of Metals and Alloys under Extreme Impacts, Ufa University of Science and Technology, 450076 Ufa, Russia; alfizg66@gmail.com; 2 Institute of Electrical Engineering, Ufa University of Science and Technology, 450076 Ufa, Russia; frg1982@mail.ru (R.F.); sabitovazat1669@yandex.ru (A.S.); 3 N.N. Blokhin National Medical Research Center of Oncology (N.N. Blokhin NMRCO), Ministry of Health of the Russian Federation, 115478 Moscow, Russia; n_anisimova@list.ru (N.A.); kisele@inbox.ru (M.K.); 4 Institute of Medicine, RUDN University, 117198 Moscow, Russia

**Keywords:** biomimetic coating, plasma electrolytic oxidation, titanium alloy, bioactive implants, anticancer therapy, corrosion, local drug delivery

## Abstract

**Background and Objectives:** Currently, the development of local drug delivery systems for the treatment of cancer patients is a pressing issue. Such systems allow for the targeted delivery of anticancer drugs directly to the tumor site, ensuring prolonged drug release or reducing the risk of recurrence after tumor removal, minimizing the impact on healthy tissues and thereby reducing the overall toxic load on the body. This work is devoted to evaluating the prospects of using scaffolds based on low-modulus titanium Ti-15Mo alloy with a biomimetic coating as a platform for the local administration of the cytostatic drug cisplatin into the patient’s body. **Methods:** Porous coatings were obtained by plasma electrolytic oxidation in an aqueous solution of sodium phosphate and calcium acetate with the addition of various components. The influence of coating parameters on the corrosion resistance of samples and on the antiproliferative effect of cisplatin-loaded scaffolds was evaluated. Human K562 hemoblastosis, HT116 intestinal cancer, and SKOV3 ovarian cancer cell lines were used as cell models. **Results:** It was shown that the addition of sodium phosphate (the PS type electrolyte) provides the formation of a coating with a developed system of interconnected pores characterized by an attractive combination of parameters: high porosity (17%), high pore size (3.9 μm), and considerable thickness (17.4 μm). This coating demonstrated the best corrosion resistance in a Ringer solution as compared to the other tested states. In addition, the PS coating loaded with cisplatin exhibited a pronounced cytotoxic effect on cancer cells. This effect was attributed to its ability to fix cisplatin on the surface, which slows down its release into the extracellular environment, increasing the time of its action, thereby contributing to a more effective (by more than 3 times) suppression of tumor cell proliferation compared to the action of the standard form of the drug in the form of a solution when changing the growth medium and subsequent incubation for 48 h. **Conclusions:** PS scaffolds made of low-modulus titanium alloy Ti-15Mo with a biomimetic surface in an electrolyte based on an aqueous solution of sodium phosphate and calcium acetate with the addition of sodium silicate can be used as an advanced platform for the local delivery of the cytostatic drug cisplatin, which makes them promising for application in orthopedic oncology.

## 1. Introduction

Despite the introduction of modern methods of targeted immunotherapy into clinical oncology practice, chemotherapy remains the main method of treating widespread malignant neoplasms and preventing local recurrence after radical (conditionally radical) surgical operations. At the same time, chemotherapeutic drugs, which do not have specific antitumor activity, can have a damaging effect on normal tissues, especially those with high proliferative activity (hematopoiesis, the intestinal epithelium, etc.). The half-life of chemotherapy drugs when administered intravenously is 24–72 h, and less than 0.5% of the total dose will reach the tumor [1]. In this regard, the possibility of creating an effective concentration of chemotherapy drugs is significantly limited by systemic side effects [2]. Local chemotherapy can be effective not only for the prevention of local recurrence after surgical treatment but also as palliative therapy for inoperable malignant neoplasms [3]. Therefore, drug delivery systems for local cancer therapy have been actively developed in recent decades. For these purposes, various natural and synthetic materials have been used as platforms for the local delivery of chemotherapeutic agents. In particular, various hydrogels and biodegradable polymeric materials are used as drug delivery systems [4]. Some of them have not only demonstrated their effectiveness in preclinical studies but have also been introduced into clinical practice. For example, Gliadel Wafers, biodegradable polyanhydride wafers containing the chemotherapeutic agent carmustine, are used to treat malignant glioma. However, the introduction of gels and polymeric materials into solid tumors is often associated with technical difficulties due to the density of tumor tissue, and control over their delivery and distribution is practically impossible due to the low radiopacity of the material. In addition, the degradation products of a biomaterial, even one as biocompatible as polylactide, can cause undesirable effects at the site of lactic acid release. Therefore, stable scaffolds based on medical alloys, in particular titanium-based ones, appear to be a promising alternative to hydrogels and polymeric materials, since they can be introduced into the tumor regardless of its density, and their position can be monitored using standard radiographic methods. Such scaffolds saturated with antitumor drugs are of particular interest for oncological orthopedics. In this case, such scaffolds can be used not only as fasteners or implants to replace bone tissue damaged by a tumor but also as a system for the local delivery and prolonged release of the drug to prevent local recurrence of the disease.

The criteria that materials for manufacturing such multifunctional implants must meet are as follows: high specific strength, ensuring a lighter implant design while maintaining high mechanical properties; corrosion resistance in biological fluids and biocompatibility, preventing the release of undesirable elements of the material into the human body; low elastic modulus of both the implant structure as a whole and its surface in contact with the bone to avoid stress shielding and incorrect load distribution; and biological activity of the implant to accelerate patient rehabilitation [5,6,7].

Titanium and its alloys are the most popular materials used in the manufacture of metal implants. Although the elastic modulus of pure titanium and its (α + β) alloys is significantly lower than that of cobalt–chromium alloys and steels, ranging from 100 to 105 GPa, it significantly exceeds the elastic modulus of bone (up to 40 GPa). In addition, the Ti-6Al-4V alloy, widely used in medicine, is capable of releasing large doses of V and Al ions, which are toxic to humans during prolonged use [8,9]. In this regard, the use of metastable β-titanium alloys that do not contain toxic elements and have a reduced elastic modulus is currently relevant [6]. Such alloys include Ti-15Mo, with an elastic modulus of about 80 GPa (ASTM F2066). Studies [10,11,12,13] have shown that this alloy has no toxic effect on human tissue.

However, titanium and its alloys are usually considered to be bioinert materials [14,15]. An important task is to ensure stable osteosynthesis through strong intraosseous fixation of the implant. This task can be accomplished by modifying its surface [15,16]. Much attention is currently being paid to the creation of a biomimetic implant surface that mimics the topology and chemical composition of bone, ensuring enhanced osteoconduction and osteoinduction.

Among the various methods of implant surface modification, the following main approaches can be identified: increasing roughness (shot blasting, sandblasting, nanostructuring, ultrasonic and laser treatment, acid etching, etc.) [17]; application of biomimetic coatings obtained by organic synthesis methods [18]; and application of coatings obtained mainly by physicochemical methods (physical vapor deposition, anodizing, electrolytic-plasma methods, etc.) with a structural–phase composition close to the mineral components of human bone [19]. One of the simplest, most environmentally friendly, and most suitable methods for modifying the surface of titanium implants is plasma electrolytic oxidation (PEO). Coatings obtained by this method are based on stable titanium oxides (rutile and anatase), which have high adhesion to the base material. The unique porous morphology of the coatings provides high surface roughness and a smooth change in elastic modulus from the metal implant to the bone, which also increases its biomechanical compatibility [20]. The developed surface of the PEO coating promotes the attachment of osteoblasts to the implant surface [21,22,23]. In addition, an advantage of this method is the ability to control the chemical composition of the coatings by introducing electrolyte ions (Ca, P, Si) into them, which increases the bioactivity of the surface [22,24].

Studies [12,13,25,26,27,28,29] have reported on the positive effects on developing bioactive surfaces of Ti-15Mo with enhanced properties by modifying PEO electrolytes. For example, in [12,26,29], it was shown that introducing calcium and phosphorus contents into the coating significantly affects its bioactivity, ensuring a high rate of cell proliferation. Silicon is an important element for the normal development of bones and cartilage. The authors of [30] showed that the addition of sodium silicate to the electrolyte accelerates proliferation, cell differentiation, and bone tissue mineralization. In [31], it was reported that the inclusion of silicon-containing nanoparticles in the electrolyte during PEO titanium alloy increased the corrosion resistance of the coating. Also, of particular interest is the introduction of boron and its compounds into bioactive materials for bone health and regeneration. Boron is one of the trace elements in the human body that plays an important role in many life processes, from improving bone regeneration and vascularization to providing antimicrobial protection and anti-inflammatory effects [32]. Traditional bioactive glasses and glass ceramics containing boron are currently being actively developed as bone biomaterials [33], in particular PEO coatings [34]. The introduction of boron into PEO -treated surfaces makes them promising biomimetic coatings to be used in medicine as bioactive implants [35]. In [22], the addition of boric acid to the electrolyte increased wear resistance and reduced the friction coefficient of the coating.

One of the recent burning topics relates to using porous structures to design advanced bioactive implants, in particular by loading the internal cavities with antibacterial or antitumor pharmaceuticals [7,36]. A well-developed system of PEO coatings can also be used as a controlled drug release system for local antibacterial, regenerative, and antitumor therapy [7,13,19,21]. In [13,37], the surface of Ti-15Mo was modified with the help of PEO, followed by the application of a degradable polymer with various antibiotics to the porous oxide layer. The effectiveness of such a hybrid oxide polymer coating in antibacterial therapy was demonstrated. However, studies on the application of Ti-15Mo alloy with variously modified porous surfaces to develop scaffolds for local anti-tumor therapy are scarce in the literature.

As follows from the state of the art presented above, low-modulus Ti-15Mo alloy with a biomimetic functional PEO coating has high biocompatibility, osteoinductive properties, and excellent corrosion resistance, and it is a promising material for the manufacture of implants. At the same time, its functional properties can be controlled by changing the chemical composition and morphology of the PEO-produced porous coatings. The aim of this study was to evaluate the prospects of using Ti-Mo-based scaffolds with various coatings as a platform for local administration of cytostatic drugs. An electrolyte based on an aqueous solution of sodium phosphate and calcium acetate was used. With the addition of different compounds such as sodium silicate and boric acid, various combinations and types of porous coatings have been produced.

The pores of the calcium- and phosphorus-containing PEO coatings obtained on the Ti-15Mo alloy were loaded with the anti-tumor drug, followed by in vitro testing in the tumor cell environment. It is assumed that the application of such a scaffold will reduce the risk of local recurrence after osteosynthesis in patients with primary and secondary tumors in bone tissue due to prolonged local administration of cytostatics in the postoperative period.

## 2. Materials and Methods

The material used in the study was 10 mm diameter rods made of Ti-15Mo alloy manufactured by Dynamet Carpenter, Richmond, VA, USA (Table 1).

**Figure 1:**
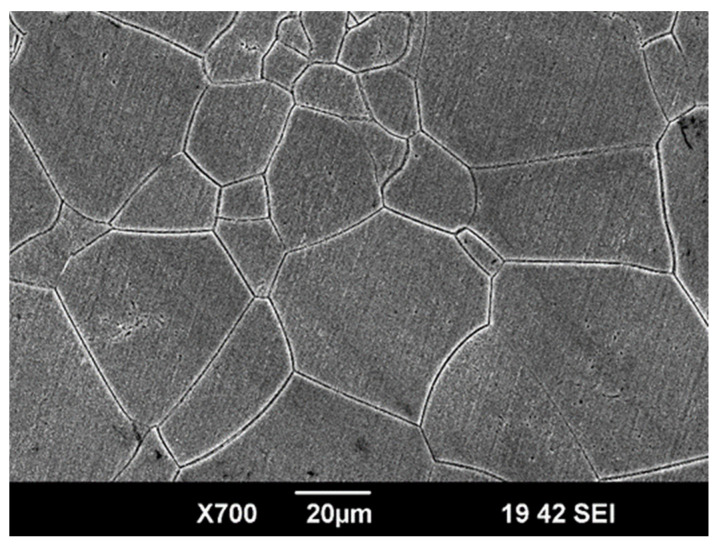
A typical SEM image presenting the microstructure of the Ti-15Mo alloy before coating deposition.

To obtain a single-phase β structure with a minimum elastic modulus, the rods were quenched in water from a temperature of 810 °C, with a holding time in the furnace of 20 min. For microstructural studies, the sample surface was sequentially ground and polished on abrasive paper with gradually decreasing grit sizes. Final polishing was performed using a silicon dioxide-based suspension on MD Chem polishing cloth (Struers China Ltd., Shanghai, China). The ground surface was then chemically etched in an aqueous solution of hydrofluoric and nitric acids (3H_2_O:1HF:1HNO_3_) for 30 s. Figure 1 shows an image of the microstructure of the hardened Ti-15Mo alloy obtained using a Jeol JSM-6390 scanning electron microscope (SEM) (Jeol, Tokyo, Japan) in secondary electron mode. The structure consists of β-grains with an average size of 50 ± 7 μm, providing reduced values of the elastic modulus.

### 2.1. Plasma Electrolytic Oxidation

Disks with a diameter of 5 mm and a thickness of 2 mm were cut from the rods, and all sides of the disks were ground and polished with abrasive paper, with final polishing on P4000 grit paper. The samples were then washed in an ultrasonic bath in water and ethyl alcohol for 5 min.

Surface modification was carried out using the PEO method on an automated installation (Ufa State Aviation Technical University, Ufa, Russia) in bipolar pulse mode with a pulse frequency of 1000 Hz at a negative pulse voltage of 40 V and a positive pulse voltage of 430 V. The process time was 5 min. An aqueous solution of sodium phosphate with calcium acetate (marked P) and with the addition of sodium silicate (PS) was used as the main electrolytes. Boric acid (B) and trilon B (T) were also added to these two electrolytes in various combinations (Table 2).

### 2.2. Coating Characterization

The pore size, thickness, porosity, and chemical composition of the PEO coatings were evaluated using images obtained with a Jeol JSM-6390 SEM (Jeol, Tokyo, Japan) equipped with an Oxford energy-dispersive analysis attachment. The thickness of the coatings was measured using SEM images of the cross-section of the samples. To determine porosity, the grid method was used to count pores larger than 1 μm. A minimum of 5 images of each state were analyzed. The roughness of the coatings was measured using a Mahr Marsurf PS10 profilometer (Mahr, Esslingen, Germany).

### 2.3. Electrochemical Tests

The electrochemical characteristics were studied using a P-5X potentiostat impedance meter (Elins LLC, Chernogolovka, Russia) in Ringer’s solution (pH = 7.4) in a 100 mL three-electrode cell with a silver chloride reference electrode (*E*_0_ = 0.222 V) and a graphite counter electrode. The electrode potential was measured for 30 min to reach a steady-state value. Next, electrochemical impedance spectroscopy (EIS) was performed in the frequency range from 100 kHz to 10 MHz relative to the steady-state electrode potential. Polarization curves were measured in the range from −600 mV to +1500 mV with a scanning rate of 0.25 mV/s relative to the electrode potential. The corrosion test duration for one sample was 6.3 h. The free corrosion potential and corrosion current were calculated using the Tafel method from the polarization curves [38]. If the Tafel section was not observed on the anodic curve of the polarization curve, then *i_corr_* was determined as an extrapolation on the abscissa axis of the intersection point of the tangent to the cathodic branch in the Tafel section and a straight line parallel to the abscissa axis drawn from the value of the free corrosion EMF *E_corr_*. The EIS results were analyzed using the ZView program from Scribner Associates. All tests were performed three times on each sample to determine the standard deviation.

### 2.4. Tests on Antiproliferative Activity

The coated Ti-Mo-based scaffolds (at least 9 disks of each type) were treated by immersion in 60% ethanol for 4 h, followed by drying in a sterile atmosphere. A volume of 5 μL of 0.4 mg/mL cisplatin (EBEWE Pharma G.M.B.H., Wien, Austria) was loaded onto the surface of each scaffold, which corresponds to 2 μg per sample. The drug-loaded scaffolds were dried in a sterile atmosphere, washed with 5 mL of Hanks’ solution (PanEco, Gorki Leninskie, Russia), and dried in a sterile atmosphere again.

The K562 human myeloid leukemia cell line, the HT116 human intestinal cancer cell line, and the SKOV3 human ovarian cancer cell line (cell line collection of the N.N. Blokhin National Medical Research Center for Oncology) were used as cell models. The choice of NT116 and SKOV3 adhesive cells as models was due to the fact that ovarian and intestinal cancer can metastasize to the bones, causing secondary bone tumors, while K562 cells were obtained from the bone marrow of a woman with chronic myeloid leukemia and therefore do not typically adhere to the matrix [39,40]. A total of 300,000 cells per well were seeded in 24-well culture plates (Corning Inc., Corning, NY, USA) in 1 μL complete growth medium based on RPMI-1640 (PanEco, Gorki Leninskie, Russia), enriched with 10% fetal bovine serum (Thermo Fisher Scientific) and 1% penicillin–streptomycin (PanEco, Gorki Leninskie, Russia) and pre-cultured for 20 h in a humidified atmosphere containing 5% CO_2_ at 37 °C. Then, the prepared drug-loaded scaffolds were added to the growth medium with cells. In the control wells, 5 μL of 0.4 mg/mL of cisplatin was added to the growth medium to produce a final concentration of 2 μg/mL (cisplatin), or nothing was added (Control). After 1 day of incubation, the growth medium was replaced in the wells with HT116 and SKOV3 cells, both those containing scaffolds and those in the control wells. The medium was not changed in the wells with K562 cells. The results were recorded in triplicate 72 h after the addition of cisplatin to the cell cultures. To assess cytotoxicity, the MTT test [41] was used, with optical density (OD) measurement at 540 nm on a Spark plate reader (Tecan, Männedorf, Switzerland).

### 2.5. Statistical Analysis

All studies were performed on at least three samples with different coatings. When analyzing the results, to identify the most effective scaffold, the antiproliferative activity of the scaffolds was compared with the effect of cisplatin added to the cell medium (cisplatin group). Differences were considered significant if the antiproliferative effect of the scaffold was more pronounced than in the cisplatin group (*p* < 0.05). For this purpose, the mean value and standard deviation of the values in each group were calculated, and the Mann–Whitney U test was performed using Statistica 10.0 (StatSoft, Tulsa, OK, USA).

## 3. Results

### 3.1. Characteristics of the PEO Coatings

**Figure 2:**
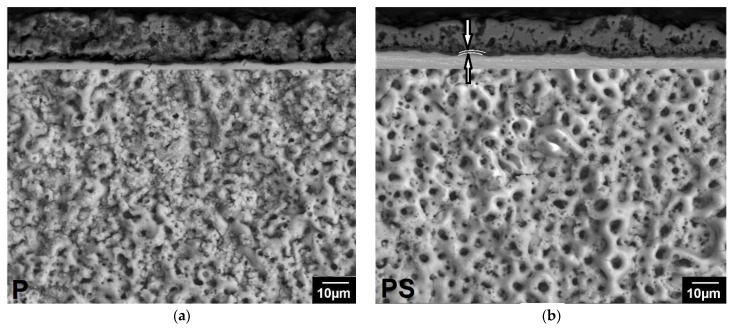

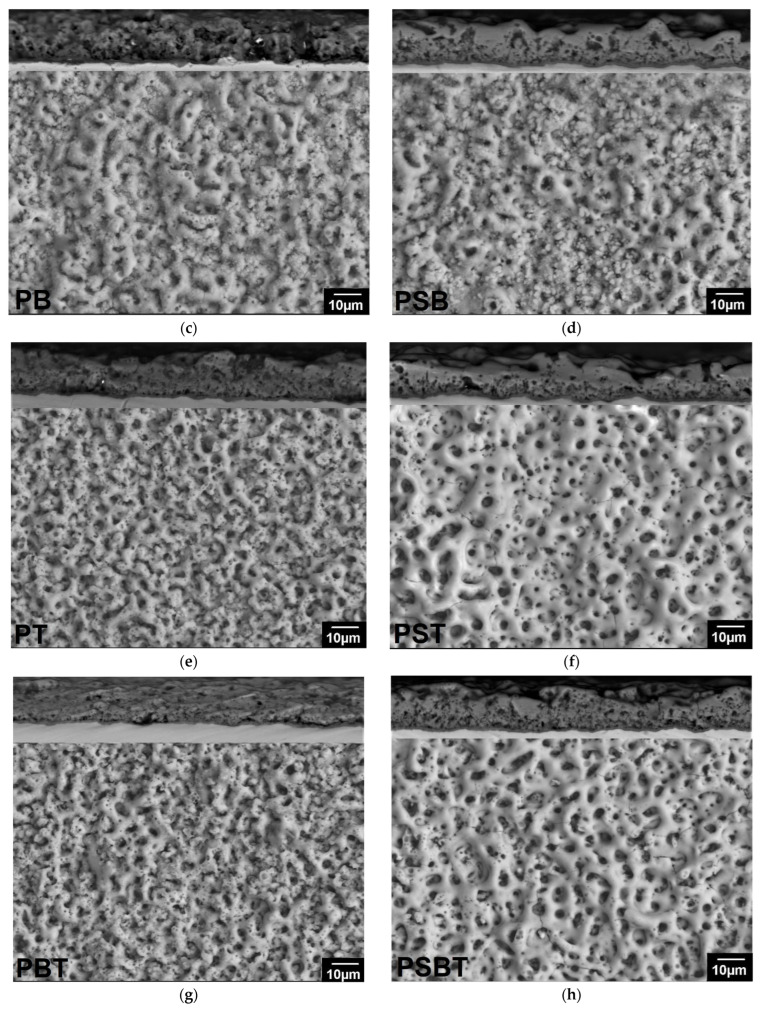
Surface morphologies of the PEO coatings produced in (**a**) P; (**b**) PS; (**c**) PB; (**d**) PSB; (**e**) PT; (**f**) PST; (**g**) PBT; and (**h**) PBST electrolytes.

The surface morphology and cross-sections of the coatings are shown in Figure 2. All coatings have a developed porous structure. The surface of the PEO coating obtained in electrolyte P is heterogeneous: rough non-porous areas or areas with pores smaller than 1 μm alternate with areas covered with pores measuring 2.7 ± 0.7 μm (Figure 2a). The cross-section (upper part of Figure 2a) reveals discontinuities between the coating and the substrate, indicating weak adhesion of the coating. The coating is loose, with many small pores. The addition of sodium silicate leads to the formation of a glossy coating surface, the pore system becomes more uniform, and large rounded pores with a size of 3.9 ± 1 μm prevail on the surface (Figure 2b), while the porosity of the coating surface increased to 17 ± 1.7%. The cross-section shows that the coating is denser than P, with no discontinuities between the coating and the substrate. The coating consists of a dense inner layer up to 2 μm thick (indicated by arrows) and a porous outer layer 17.4 ± 1.3 μm thick (Figure 2b, upper part). The pores are deep (up to 7 μm), elongated, and interconnected. The addition of boric acid to the P and PS electrolytes led to an enlargement of the pores. The porosity of the surface of the coatings obtained in the PB electrolyte without silicate increased compared to P (Figure 2c), while in the PSB electrolyte, it decreased compared to PS (Figure 2d). The surface is heterogeneous, with smoothed areas on the pore craters alternating with rough areas. During the preparation of the cross-section, the PB sample coating was partially chipped. The PSB coating was denser.

The addition of trilon B to electrolytes contributed to the formation of a thinner, more uniform coating (Table 3), an increase in the proportion of small pores (Figure 2e–h), improved adhesion, and densification of silicate-free coatings (Figure 2e,g).

Table 4 shows the elemental composition of the coatings determined by energy-dispersive analysis.

### 3.2. Electrochemical Behavior of Uncoated and PEO-Coated Samples

**Figure 3:**
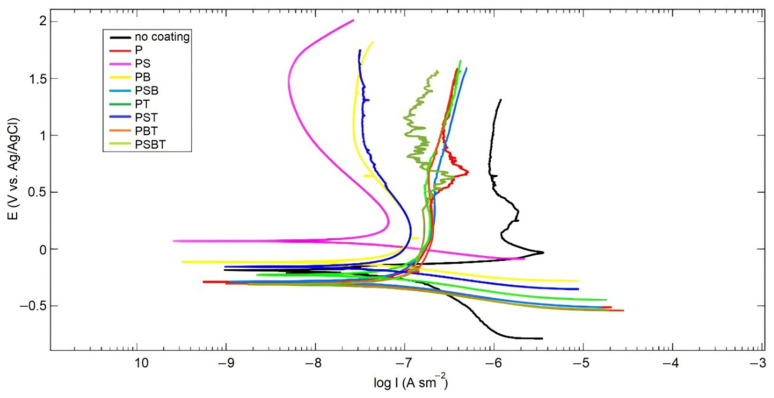
Polarization curves in Ringer’s solution for the uncoated and PEO-coated samples in various electrolyte samples.

The results of corrosion tests in the form of polarization curves are shown in Figure 3. Analysis of the polarization curves shows that Tafel regions are observed on the cathode branches of the samples. At the same time, the samples on the anode branch have passivation regions. Potentiodynamic polarization involves changing the potential of the working electrode and measuring the current. This method provides useful information on the susceptibility to corrosion in a given environment (thermodynamic information—free corrosion potential *E*_corr_) and the corrosion rate (kinetic information—corrosion current *i*_corr_). *E*_corr_ indicates corrosion susceptibility, while *i*_corr_ determines the average corrosion rate over the studied sample area. Generally, higher *E*_corr_ and lower *i*_corr_ indicate higher corrosion resistance and better anticorrosive properties.

Table 5 presents the results of calculating corrosion parameters in the form of free corrosion EMF *E_corr_*, corrosion current *i_corr_*, and polarization resistance *R_p_*.

The calculated corrosion parameters show that the highest corrosion current is observed in the uncoated sample (WC). The lowest corrosion current is observed in the sample treated in PS electrolyte; moreover, this sample has the most passive surface, as indicated by the shift in the free corrosion potential *E_corr_* towards a higher potential. The addition of boric acid to the silicate-free electrolyte P reduced the corrosion current, while in the PS electrolyte, it was increased. The addition of Trilon B to the electrolytes leads to an increase in corrosion currents. The values of polarization resistances *R_p_* are consistent with the values of corrosion currents.

**Figure 4:**
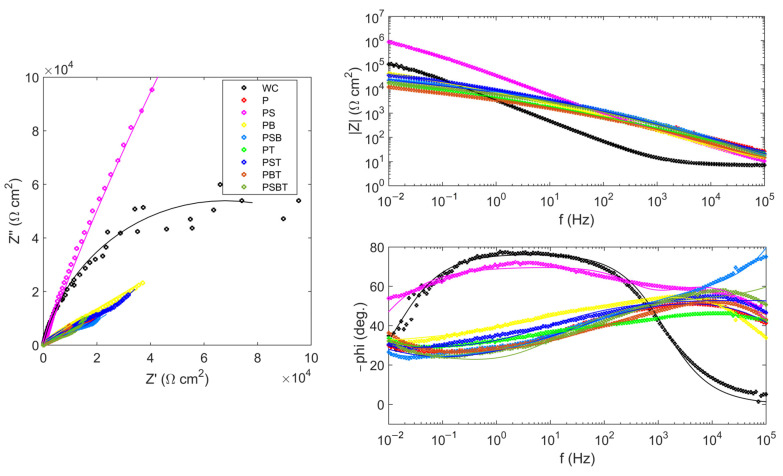
Nyquist and Bode plots for the EIS of the uncoated and PEO-coated samples in various electrolyte samples in Ringer’s solution.

**Figure 5:**
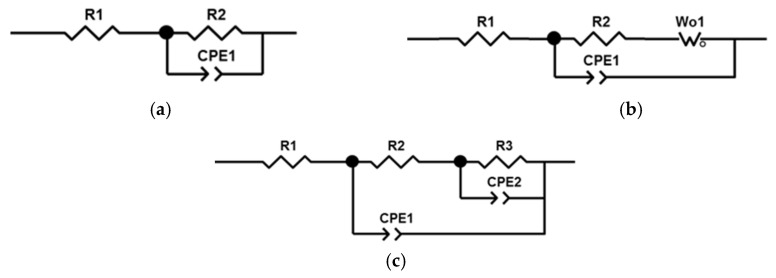
Equivalent circuits used for fitting of EIS results: (**a**) for WC sample; (**b**) for P, PB, PSB, PT, PST, PBT, and PSBT samples; (**c**) for PS sample.

Figure 4 shows the impedance spectra of the samples. The spectrum of the uncoated sample was approximated by a Randles circuit with a single time constant (Figure 5a). Resistance R1 represents the resistance of the electrolyte; it is the same for all samples and is 7.54 ± 0.06 Ω. The pair of resistances R2-CPE1 have the physical meaning of the active resistance to charge transfer and the capacitance of the double electric layer, respectively. The value of resistance R2 in the untreated sample is higher than in the samples with PEO coating. This is due to the fact that a thin natural oxide layer with high resistance is formed on the surface of the substrate due to the small number of defects, as indicated by the high value of the CPE1-*n* parameter.

The impedance spectra of samples with PEO coatings were approximated by two different circuits with two time constants. The difference in the circuits is due to the different structure, morphology, and corrosion processes occurring on the surface of PEO coatings. The impedances of the remaining samples were approximated by a circuit (Figure 5b) containing a Warburg element that reflects the diffusion processes occurring in the pores of the coating. Diffusion processes manifest themselves as an increase in the impedance of the system at an angle of 45° to the real axis with a decrease in frequency. At high frequencies, the influence of Warburg impedance is negligible, so the first semicircle is approximated by the pair *R2–CPE1*. From the large values of the Warburg impedance parameters *Wo-R* and the time constant *Wo-T*, it follows that diffusion processes experience great difficulties in the coating obtained in the PB electrolyte. The CPE1 element in all equivalent circuits can be considered in the first approximation as the capacitance of a double electric layer, which determines the thickness of the protective coating *h~1/CPE1-Q*. The highest value of *CPE1-Q* in the untreated sample indicates a thin oxide layer. In samples after PEO treatment, the value of *CPE1-Q* is lower, which qualitatively indicates a greater thickness of the oxide layer.

The impedance of the PS sample was approximated by a classic ladder circuit (Figure 5c). This hodograph contains two time constants, which indicates the presence of an internal barrier layer and an external porous layer. In the circuit, elements R2 and CPE1 correspond to the resistance and capacitance of the external porous layer of the PEO coating. The pair of elements *R3–CPE2* describes charge transfer during corrosion testing. Here, R3 is the charge transfer resistance, and element CPE2 can be considered, as a first approximation, to be the capacitance of the double electric layer at the phase boundary between the internal barrier layer and the electrolyte. The higher value of resistance R3 compared to R2 and of CPE2-*n* compared to CPE1-*n* indicates the absence of a significant number of defects in the structure of the internal barrier layer, which becomes the main contribution to the protective characteristics of the PEO coating. The results of calculating the parameters of equivalent circuits are presented in Table 6.

The sample treated in PS electrolyte has the best corrosion characteristics. The addition of component T, Trilon B, leads to a slight deterioration in corrosion properties.

### 3.3. Bioactive Properties

**Figure 6:**
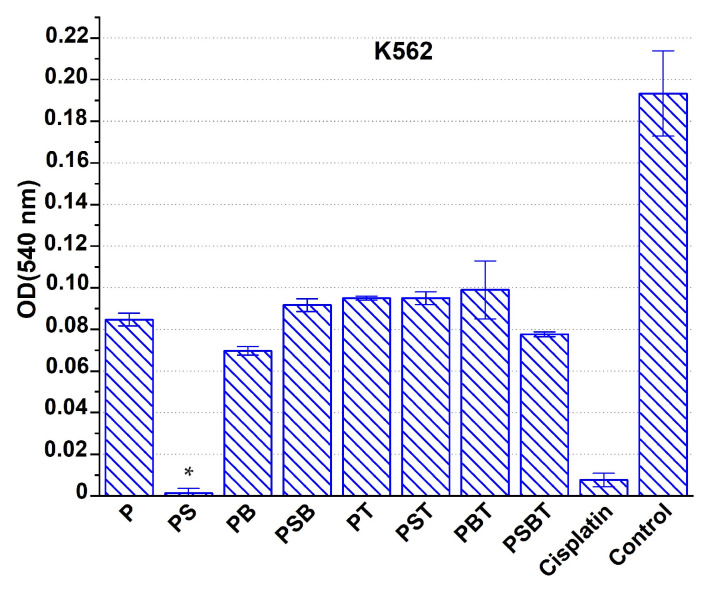
Suppression of the viability of K562 cells under the action of cisplatin-loaded Ti-15Mo-based scaffolds with various coatings and with this drug added directly to the growth medium (cisplatin), compared with the intact control. Incubation time: 72 h. * Significant cytotoxic effect compared to the cisplatin group, *p* < 0.05.

In experiments on the K562 cell line model, scaffolds with cytostatic agents were added to the cell incubation medium without changing the medium composition for the next 72 h. As a result, it was found that the addition of cisplatin loaded onto scaffolds of all types to the culture medium had a pronounced cytotoxic effect compared to the intact control (Figure 6). However, compared to the control with cisplatin directly added to the growth medium, only PS scaffold-based constructs had a similar cytotoxic effect in terms of intensity.

**Figure 7:**
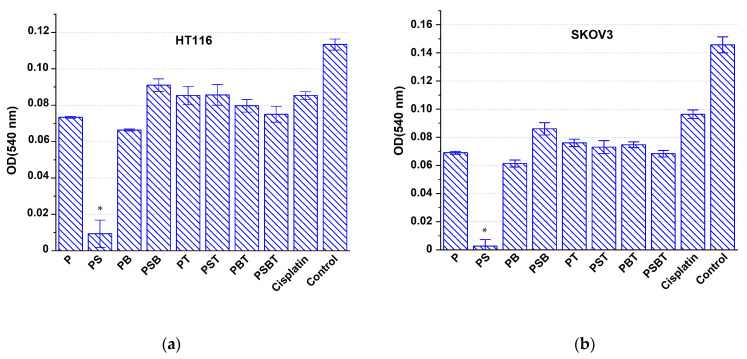
Antiproliferative effect on HT116 (**a**) and SKOV3 (**b**) cells of Ti-15 Mo-based scaffolds with different coatings loaded with cisplatin, compared with the effect of cisplatin in solution (cisplatin) and intact control after complete replacement of the medium after 1 day and subsequent incubation for 48 h. * Significant cytotoxic effect compared to the cisplatin group, *p* < 0.05.

The next series of experiments on HT116 and SKOV3 cell models was characterized by replacement of the growth medium 1 day after the introduction of cisplatin-loaded scaffolds. In this case, the suppression of cell viability in the cisplatin control was significantly weaker compared to the effect on K562 cells, since most of the preparation was removed along with the medium. The most pronounced antiproliferative effect on the growth of HT116 and SKOV3 cell lines was observed under the influence of cisplatin-containing structures with a PS structure (Figure 7). PB-coated scaffolds loaded with cisplatin also had a noticeable cytotoxic effect under the conditions of this experiment.

## 4. Discussion

General chemotherapy or radiation therapy for the treatment of cancer or to prevent recurrence of the disease after surgical intervention to remove a tumor has a systemic toxic effect on the body, affecting not only cancer cells but also cells in healthy tissue, which leads to the intoxication of the patient’s body. In this regard, the development of local drug delivery systems for the treatment of cancer patients is becoming increasingly relevant. Such systems allow anticancer drugs to be delivered directly to the tumor site, ensuring prolonged drug release or reducing the risk of recurrence after tumor removal, reducing the impact on healthy tissues and thereby reducing the overall toxic load on the body [42,43].

To date, various options have been developed for the local delivery of anticancer drugs based on hydrogels or biodegradable polymer materials [44,45]. However, biodegradable platforms for the delivery of chemotherapy drugs have not yet become widely used in clinical practice. They are mainly used for the prevention of recurrence of resected brain tumors [46]. Meanwhile, the problem of preventing tumor recurrence remains very important in the field of oncological orthopedics. This is especially relevant after conditionally radical surgery for osteogenic sarcoma, as there is evidence that 30–40% of patients with localized osteosarcoma subsequently develop recurrence (including local recurrence and distant metastases), which mediates low survival rates, corresponding to 23–29% [47]. After resection of bones destroyed by the tumor, osteoconstructive surgery and arthrodesis are required. This includes the use of metal structures. Therefore, it appears that combining metal structures for onco-orthopedics with properties such as mechanical strength, reduced elastic moduli, and a prolonged local antitumor effect can provide a competitive advantage over existing analogs in clinical practice.

In this work, the low-modulus β-titanium alloy Ti-15Mo was used to create scaffolds for local antitumor therapy. The surface of the samples was modified using the PEO method. Eight calcium- and phosphorus-containing coatings with a high Ca/P ratio and different chemical compositions and morphological characteristics were obtained by varying the electrolyte components. The samples were loaded with the antitumor drug cisplatin. Three lines of human tumor cells of different origins were used as cell models.

In experiments on the K562 cell line model, only PS samples had a real cytotoxic effect comparable to the control group. It can be assumed that the lower cytotoxic effect of the other designs was due to the strong binding of part of the cytostatic agent to the surface of the samples, which led to a reduced concentration of the drug in the medium. Alternatively, after the initial complete dissolution of cisplatin, its re-sorption by the scaffold surface was observed. Compared to other samples, scaffolds with PS and PB coatings had the most active effect.

The design of the next series of experiments on cell models of the HT116 and SKOV3 lines mimics in vivo conditions, where the environment around the implant must be constantly renewed by blood circulation and lymph flow, ensuring clearance of the administered cytostatic drug. It was shown that the suppression of cancer cells in the control group with cisplatin was relatively small, probably due to the removal of most of the drug along with the first portion of the growth medium. This proves that the dose of the drug remaining in the medium is insufficient for effective suppression of tumor cell culture growth. In contrast, the use of all the scaffolds studied contributed to a more intense cytotoxic effect compared to the control with cisplatin. This proves that the coating contributed to the preservation of a significant part of the drug near the titanium sample even after washing, ensuring the prolongation of the effect of cisplatin due to the prolonged release and maintenance of an effective cytotoxic concentration in the growth medium.

Experiments with different cell models were aimed at providing a more comprehensive description of the antiproliferative activity of the developed scaffolds. During potential in vivo implantation of the scaffold, both scenarios described (with different cell models) could occur near the implant, depending on the extent of blood and lymph flow disturbances. Specifically, after surgery, a lack of tissue clearance can occur, leading to a cumulative accumulation of the drug released from the coatings of the metal structures (a situation similar to experiments on K562 cells), as well as rapid renewal of the environment, which will be accompanied by drug removal from the tissues near the implant (a situation similar to experiments on adherent cells). Therefore, it was important to evaluate how the properties of coated scaffolds would be manifested under these different conditions and to try to assess their clinical prospects.

**Figure 8:**
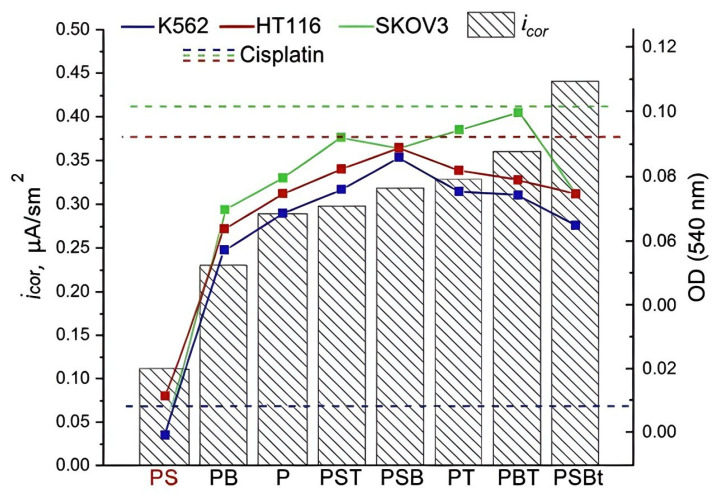
Corrosion properties and antiproliferative effect on tumor cells of the K562, HT116, and SKOV3 lines of scaffolds with different types of PEO coatings loaded with cisplatin.

The data obtained show that scaffolds with the studied coatings promoted the fixation of cisplatin on their surface. This slowed down its release into the extracellular environment, prolonging its exposure time, which contributed to more effective inhibition of tumor cell proliferation compared to the effect of the standard form of the drug as a solution. PS scaffolds loaded with cisplatin showed the greatest effectiveness, which is probably due to the characteristics of the PEO coating. It has been established that the addition of sodium silicate to the electrolyte contributes to the formation of a dense coating with a developed system of interconnected pores that have good adhesion to the substrate. At the same time, the samples obtained in the PS electrolyte have the highest coating surface porosity, the largest pore diameter and depth, and the greatest coating thickness (Figure 2, Table 3) compared to other samples obtained in electrolytes with the addition of sodium silicate. It is likely that this combination of coating parameters allows PS scaffolds to be loaded with a large amount of the drug, contributing to its longer release and thus ensuring its prolonged effect. In addition to superior cytostatic effect, PS samples demonstrate higher corrosion resistance (Table 5, Figure 8).

The results of this study indicate that the developed titanium scaffolds with a PS-modified surface can be saturated with cisplatin, ensuring its prolonged release. This is evidenced by the preservation of cytotoxic activity against SKOV3 and HT116 tumor cells 48–72 h after replacing the growth medium in the presence of a cisplatin-loaded scaffold. It seems obvious that this effect was achieved due to the initial adsorption of the drug by the PS-modified surface during loading, followed by slow release in an aqueous medium. Thus, unlike existing analogs, the proposed metal structures can be used not only as bone plates for osteosynthesis. According to the collected data, the PS samples allow prolonging the persistence of the cytostatic drug in the tumor cell incubation medium, which will mediate a reduction in the risk of tumor growth activation in the area of their implantation for the osteosynthesis area.

Note that, previously [7,48], commercially pure titanium was used as a substrate for applying multilayer coatings using 5-Fluorouracil (5-FU) loaded in calcium phosphate, which is encapsulated in poly(lactic-co-glycolic acid) (PLGA/CaP) for controllable drug release. The effective cytotoxicity of the drug was demonstrated due to its prolonged release. The novelty of the present work relates to the investigation of the drug delivery potential of a metastable Ti-15Mo alloy containing no potentially toxic elements and with a significantly lower elastic modulus than that of pure titanium or the popular Ti-6Al-4V alloy (80 and ~110 GPa, respectively). In addition, an important novel aspect of this work relates to the experiment simulating blood and lymph flow in the body by changing the growth environment.

To sum up, PS scaffolds made of low-modulus titanium alloy Ti-15Mo with a biomimetic coating obtained by PEO in an electrolyte based on an aqueous solution of sodium phosphate and calcium acetate with the addition of sodium silicate can be used as a platform for the local delivery of the cytostatic drug cisplatin, which makes it promising for applications in orthopedic oncology.

## Figures and Tables

**Table 1:** Chemical composition of Ti-15Mo according to the material certificate (wt.%).

Ti	Mo	O	Fe	C	N
balance	15.2	0.16	0.02	0.008	0.10

**Table 2:** Sample codes corresponding to electrolyte compositions.

Sample Code	Electrolyte Composition
P	15 g L^−1^ Na_3_PO_4_∙12 H_2_O + 25 g L^−1^ Ca(CH_3_COO)_2_·1 H_2_O
PS	15 g L^−1^ Na_3_PO_4_∙12 H_2_O + 25 g L^−1^ Ca(CH_3_COO)_2_·1 H_2_O + 5 g L^−1^ Na_2_SiO_3_∙12 H_2_O
PB	15 g L^−1^ Na_3_PO_4_∙12 H_2_O + 25 g L^−1^ Ca(CH_3_COO)_2_·1 H_2_O + 1 g L^−1^ H_3_BO_3_
PSB	15 g L^−1^ Na_3_PO_4_∙12 H_2_O + 25 g L^−1^ Ca(CH_3_COO)_2_·1 H_2_O + 5 g L^−1^ Na_2_SiO_3_∙12 H_2_O + 1 g L^−1^ H_3_BO_3_
PT	15 g L−1 Na_3_PO_4_∙12 H2O + 25 g L−1 Ca(CH_3_COO)_2_·1 H_2_O + 3.4g L^−1^ + C_10_H_14_N_2_Na_2_O_8_
PST	15 g L^−1^ Na_3_PO_4_∙12 H_2_O + 25 g L^−1^ Ca(CH_3_COO)_2_·1 H_2_O + 5 g L^−1^ Na_2_SiO_3_∙12 H_2_O + C_10_H_14_N_2_Na_2_O_8_
PBT	15 g L^−1^ Na_3_PO_4_∙12 H_2_O + 25 g L^−1^ Ca(CH_3_COO)_2_·1 H_2_O + 1 g L^−1^ H_3_BO_3_ + C_10_H_14_N_2_Na_2_O_8_
PSBT	15 g L^−1^ Na_3_PO_4_∙12 H_2_O + 25 g L^−1^ Ca(CH_3_COO)_2_·1 H_2_O + 5 g L^−1^ Na_2_SiO_3_∙12 H_2_O + 1 g L^−1^ H_3_BO_3_ + C_10_H_14_N_2_Na_2_O_8_

**Table 3:** Morphological characteristics of the PEO coatings.

Sample Code	Pore Size, µm	Porosity, %	Coating Thickness, µm	Roughness Ra, µm
P	2.7 ± 0.7	4 ± 0.5	17.2 ± 0.9	1.49 ± 0.07
PS	3.9 ± 1	17 ± 1.7	17.4 ± 1.3	1.20 ± 0.05
Pb	3.2 ± 0.8	12 ± 0.7	18.8 ± 1	1.41 ± 0.07
PSB	4 ± 1.4	5 ± 0.7	16.4 ± 1.1	1.36 ± 0.13
Pt	3.2 ± 0.8	10 ± 1.2	15.7 ± 1.1	1.03 ± 0.08
PSt	3.5 ± 1	15 ± 1.3	11.6 ± 1.4	1.36 ± 0.06
PBt	2.9 ± 0.8	9 ± 0.8	14.3 ± 0.5	1.19 ± 0.05
PSBt	3.6 ± 1	16 ± 0.9	13.6 ± 0.9	1.18 ± 0.05

**Table 4:** The elemental composition of the coatings.

Sample Code	Content of the Elements in the Coating (wt%)						
	O	Ti	Si	Ca	Na	P	Ca/P
P	39 ± 0.7	32.3 ± 0.9	-	18.8 ± 1.9	0.7 ± 0.1	8.4 ± 0.6	2.2 ± 0.2
PS	41.9 ± 0.9	31.5 ± 1.0	5.1 ± 0.4	15.9 ± 0.2	0.5 ± 0.2	5.1 ± 0.5	3.1 ± 0.2
PB	40.0 ± 0.8	32.4 ± 0.9	-	18.1 ± 0.6	0.4 ± 0.1	9.0 ± 0.6	2.0 ± 0.3
PSB	42.2 ± 0.7	31.9 ± 1.1	4.3 ± 0.2	15.8 ± 0.6	0.4 ± 0.2	5.5 ± 0.9	2.87 ± 0.1
PT	53.5 ± 0.6	23.3 ± 0.7	-	12.0 ± 0.7	0.5± 0.1	9.1 ± 0.6	1.3 ± 0.1
PST	46.5 ± 0.6	31.6 ± 0.6	6.2 ± 0.9	9.3 ± 0.5	0.9 ± 0.2	3.4 ± 0.3	2.7 ± 0.3
PBT	40.2 ± 0.7	29.5 ± 0.7	-	15.9 ± 0.3	0.4 ± 0.1	13.8 ± 0.7	1.2 ± 0.1
PBST	41.0 ± 0.9	35.05	4.9 ± 0.5	15.3 ± 0.5	0.5 ± 0.1	3.28 ± 0.3	4.7 ± 0.2

**Table 5:** Results of potentiodynamic corrosion tests in Ringer’s solution for the uncoated and PEO-coated in various electrolyte samples.

Sample Code	*E_corr_*, V	*i_corr_*, μA/sm^2^	*R_p_*, kΩ·sm^2^
WC	−0.186 ± 0.12	0.55 ± 0.14	69.60 ± 19.34
P	−0.290 ± 0.07	0.29 ± 0.07	73.30 ± 16.95
PS	0.069 ± 0.03	0.11 ± 0.01	84.20 ± 3.48
PB	−0.115 ± 0.03	0.22 ± 0.02	67.90 ± 4.51
PSB	−0.287 ± 0.17	0.32 ± 0.03	61.20 ± 13.05
PT	−0.229 ± 0.01	0.33 ± 0.01	56.90 ± 2.86
PST	−0.156 ± 0.06	0.30 ± 0.02	66.50 ± 9.86
PBT	−0.305 ± 0.13	0.37 ± 0.11	55.00 ± 10.36
PSBT	−0.313 ± 0.02	0.44 ± 0.02	52.90 ± 14.29

**Table 6:** EIS fit results of the parameters of the equivalent circuits for the uncoated and PEO-coated samples tested in Ringer’s solution.

Sample Code	R2, kΩ·sm^2^	R3, MΩ·sm^2^	CPE1-Q, μF^n−1^·sm^−2^	CPE1-n	Wo-R, kΩ·sm^2^	Wo-T, s	Wo-P	CPE2-Q, μF^n−1^·sm^−2^	CPE2-n
WC	135.75 ± 3.15	–	59.45 ± 0.39	0.85 ± 0.001	–	–	–	–	–
P	1.28 ± 0.77	–	10.93 ± 1.05	0.62 ± 0.008	34.92 ± 2.46	82.2 ± 16.3	0.24 ± 0.02	–	–
PS	0.71 ± 0.07	2.36 ± 0.09	8.79 ± 0.15	0.69 ± 0.001	–	–	–	0.49 ± 0.11	0.93 ± 0.03
PB	8.81 ± 0.51	–	26.49 ± 0.34	0.61 ± 0.001	130.40 ± 46.25	259.3 ± 256.2	0.38 ± 0.01	–	–
PSB	7.26 ± 0.47	–	16.64 ± 0.37	0.58 ± 0.003	44.62 ± 6.82	120.3 ± 50.2	0.35 ± 0.01	–	–
PT	4.91 ± 0.39	–	46.16 ± 0.77	0.54 ± 0.002	35.43 ± 3.27	77.9 ± 17.4	0.38± 0.01	–	–
PST	10.03 ± 1.09	–	19.19 ± 0.28	0.58 ± 0.002	76.34 ± 7.69	104.3 ± 27.57	0.36 ± 0.007	–	–
PBT	3.32 ± 0.34	–	40.01 ± 0.74	0.55 ± 0.002	18.21 ± 1.18	44.31 ± 5.74	0.39 ± 0.008	–	–
PSBT	5.14 ± 0.46	–	27.45 ± 2.18	0.60 ± 0.002	27.45 ± 2.18	56.34 ± 9.92	0.38 ± 0.008	–	–

## Data Availability

The original contributions presented in this study are included in the article. Further inquiries can be directed to the corresponding authors.

## Abbreviations

The following abbreviations are used in this manuscript:

PEO	Plasma Electrolytic Oxidation
SEM	Scanning Electron Microscope
EIS	Electrochemical Impedance Spectroscopy
OD	Optical Density
